# Chlamydomonas ATX1 is essential for Cu distribution to multiple cupro‐enzymes and maintenance of biomass in conditions demanding cupro‐enzyme‐dependent metabolic pathways

**DOI:** 10.1002/pld3.383

**Published:** 2022-02-03

**Authors:** Keegan L. J. Pham, Stefan Schmollinger, Sabeeha S. Merchant, Daniela Strenkert

**Affiliations:** ^1^ Department of Plant and Microbial Biology University of California Berkeley California USA; ^2^ California Institute for Quantitative Biosciences University of California Berkeley California USA; ^3^ Department of Molecular & Cell Biology University of California Berkeley California USA

## Abstract

Copper (Cu) chaperones, of which yeast ATX1 is a prototype, are small proteins with a Cu(I) binding MxCxxC motif and are responsible for directing intracellular Cu toward specific client protein targets that use Cu as a cofactor. The *Chlamydomonas reinhardtii* ATX1 (CrATX1) was identified by its high sequence similarity with yeast ATX1. Like the yeast homologue, CrATX1 accumulates in iron‐deficient cells (but is not impacted by other metal‐deficiencies). N‐ and C‐terminally YFP‐ATX1 fusion proteins are distributed in the cytoplasm. Reverse genetic analysis using artificial microRNA (amiRNA) to generate lines with reduced CrATX1 abundance and CRISPR/Cpf1 to generate *atx1* knockout lines validated a function for ATX1 in iron‐poor cells, again reminiscent of yeast ATX1, most likely because of an impact on metalation of the multicopper oxidase FOX1, which is an important component in high‐affinity iron uptake. We further identify other candidate ATX1 targets owing to reduced growth of *atx1* mutant lines on guanine as a sole nitrogen source, which we attribute to loss of function of UOX1, encoding a urate oxidase, a cupro‐enzyme involved in guanine assimilation. An impact of ATX1 on Cu distribution in *atx1* mutants is strikingly evident by a reduced amount of intracellular Cu in all conditions probed in this work.

## 1 INTRODUCTION

Copper (Cu) is an important trace element in all kingdoms of life, because it serves as an essential cofactor in enzymes that participate in diverse metabolic pathways, from photosynthesis and respiration to oxidative stress protection and iron acquisition. Although a particular quota of Cu is required for fully functioning metabolism, excess intracellular Cu ions may result in damage of macromolecules through redox or oxygen chemistry with negative consequences for the cell. Cu assimilation and sequestration is therefore tightly regulated by orchestrated function of Cu transporters, ligands, and chaperones.

A general pathway for Cu metabolism has been developed based on discoveries in several systems. Cu enters the cell through a high affinity Cu(I) uptake system that includes CTR/COPT family proteins (Puig & Thiele, 2002). After entry, cytosolic chaperone proteins (of which ATX1 is a prototype) are responsible for subsequent Cu transfer to key metabolic Cu proteins (Culotta et al., 1997; Rosenzweig & O'Halloran, 2000; Shi et al., 2021). More recently, GSH is implicated as a Cu ion carrier between the importers and metallochaperones (Miras et al., 2008).

In *Saccharomyces cerevisiae*, where Cu metabolism is well dissected, three Cu chaperones compete for Cu entering the cell, delivering them to their specific targets (Valentine & Gralla, 1997). yATX1 is a small protein (73 aa) that delivers Cu to the trans‐Golgi network for incorporation into membrane‐bound and secreted Cu proteins (Lin & Culotta, 1995). yATX1 was discovered originally as a suppressor of oxygen toxicity damage in yeast strains lacking SOD1 function, hence its name Atx for antioxidant (Lin & Culotta, 1995). The Cu binding motif, MXCXXC, is present also in many ATP‐dependent Cu exporters (Cu P‐type ATPases). yATX1 transfers Cu from the inner plasma membrane to the secretory pathway where Cu P‐type ATPases such as Ccc2 are localized for sequestering Cu into the secretory pathway (Hung et al., 1997; Lin et al., 1997). Ccc2 directly interacts with ATX1, enabling Cu transfer to Ccc2 for translocating Cu coupled to ATP hydrolysis (Arnesano et al., 2002; Pufahl et al., 1997). Notably, all Cu‐dependent enzymes from the secretory pathway are loaded with Cu ions in the Golgi. One such enzyme is the cupro‐protein FET3, a plasma membrane spanning multicopper ferroxidase, which is required for high‐affinity iron uptake in yeast (Lin et al., 1997). Accordingly, yeast *atx1* mutants are unable to load Cu into the active site of FET3, resulting in defective iron uptake and iron deficiency (Lin et al., 1997). Yeast *ctr1* mutants defective in Cu import and yeast *ccc2* mutants defective in transferring Cu to the secretory pathway also display an iron deficiency phenotype that can be rescued by excess Cu (Askwith & Kaplan, 1998; Dancis et al., 1994).

Atx‐like Cu chaperones are found with high sequence similarity in most eukaryotes, including mammals, algae, and land plants. The human ATX1 homologue (*HAH1*) was shown to rescue the growth defect of yeast *atx1* mutants in iron‐poor medium by restoring Cu incorporation into newly synthesized FET3 (Klomp et al., 1997). HAH1 directly transfers Cu ions to the Cu P‐type ATPases ATP7A and ATP7B, which are involved in Cu delivery to the secretory pathway (Banci et al., 2005). Two ATX1 homologues named Cu CHaperone (CCH) and AtATX1 have been analyzed from *Arabidopsis*. Both proteins, CCH as well as AtATX1, complement the iron conditional growth defect of yeast *axt1* mutants (Himelblau et al., 1998; Puig et al., 2007). Direct interaction with the plant Cu P‐type ATPase RAN1 was established for AtATX1 (Puig et al., 2007). Mutants defective in AtATX1 are hypersensitive to both Cu excess and Cu deficiency conditions (Shin et al., 2012), indicating the importance of this protein for Cu homeostasis.

**FIGURE 1:**
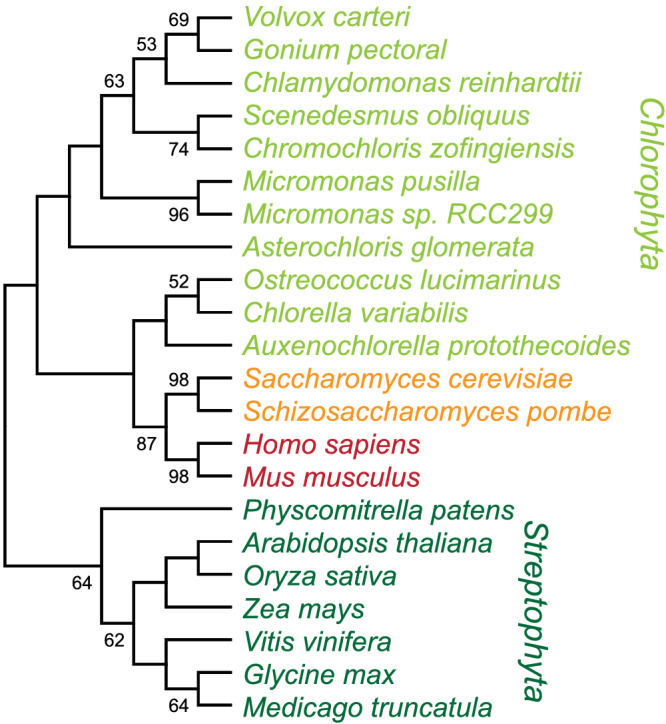
ATX1 is a conserved and widespread protein. The evolutionary history was inferred by using the maximum likelihood method and JTT matrix‐based model (Jones et al., 1992). The bootstrap consensus tree inferred from 1000 replicates (Felsenstein, 1985) is taken to represent the evolutionary history of the taxa analyzed (Felsenstein, 1985). When the percentage of replicate trees in which the associated taxa clustered together in the bootstrap test (1000 replicates) exceeded 50%, they are shown next to the branches. This analysis involved 22 amino acid sequences. There were a total of 90 positions in the final dataset. Evolutionary analyses were conducted in MEGA X (Kumar et al., 2018)

Like other algae, the eukaryotic green alga *Chlamydomonas reinhardtii*, referred to as Chlamydomonas hereafter, has a reduced Cu quota, perhaps because of the lack of a Cu/ZnSOD (Asada et al., 1977; Omelchenko et al., 2010). The genome of Chlamydomonas encodes a single ATX1 homologue, CrATX1 (Figure 1). Transcriptional profiling indicates that Cr*ATX1* expression is increased in response to iron deficiency in Chlamydomonas along with other components of Fe assimilation like *FOX1*, *FTR1*, *FER1*, and *FEA1* (La Fontaine et al., 2002). An orthologous function to yeast ATX1 and human HAH1 was suggested based on functional complementation of the yeast *ATX1* strain and the relationship of multicopper oxidase CrFOX1 to yFET3 (La Fontaine et al., 2002; Urzica et al., 2012).

In this work, we extend the original study to document selective *ATX1* expression and protein accumulation in response to iron and nitrogen nutrition (but not Cu or Zn), localize YFP‐tagged ATX1 to the cytoplasm, and assess function in Chlamydomonas by reverse genetics under conditions where cupro‐proteins are required: Fe limitation and guanine as a sole N‐source, requiring, respectively, cupro‐enzymes FOX1 and UOX1 encoding a multicopper Fe(II) oxidase and urate oxidase. We conclude that CrATX1 is the cytosolic Cu chaperone responsible for Cu delivery from CTR‐like Cu importers and/or GSH at the plasma membrane to the secretory pathway. But surprisingly, our work indicates that ATX1 might have an additional, more general role in copper homeostasis, as *atx1* mutants have a significantly reduced copper content in all conditions that were interrogated in this work.

## 2 EXPERIMENTAL

### 2.1 Strains and culture conditions

An miRNA targeting Chlamydomonas *ATX1* was designed according to (Molnár et al., 2009; Schmollinger et al., 2010) using the WMD3 tool at http://wmd3.weigelworld.org/. Resulting oligonucleotides ATX1amiFor: ctagtATGGTCGTCAAGACGGCGAAAtctcgctgatcggcaccatgggggtggtggtgatcagcgctaTTTCCCCGTCTTGACGACCATg and ATX1amiRev: ctagcATGGTCGTCAAGACGGGGAAAtagcgctgatcaccaccacccccatggtgccgatcagcgagaTTTCGCCGTCTTGACGACCATa (uppercase letters representing miRNA*/miRNA sequences) were annealed by boiling and slowly cooling down in a thermocycler and ligated into SpeI‐digested pMS539 (Schmollinger et al., 2010), yielding pDS16. pDS16 was linearized by digestion with HindIII and transformed into Chlamydomonas strain CC‐4351 by vortexing with glass beads. Mutants in *atx1* (amiRNA strains and CRISPR/Cpf1‐mediated KO strains) and *C. reinhardtii* strain CC‐4533 were grown in tris‐acetate‐phosphate (TAP) growth medium with constant agitation in an Innova incubator (180 rpm, New Brunswick Scientific, Edison, NJ) at 24°C in continuous light (90 μmol m^−2^ s^−1^), provided by cool white fluorescent bulbs (4100 K) and warm white fluorescent bulbs (3000 K) in the ratio of 2:1, unless stated otherwise. TAP medium with or without iron or nitrogen was used with revised trace elements (Special K) instead of Hutner's trace elements according to Kropat et al. (2011). For all experiments with *atx1* amiRNA strains, a modified TAP medium (TAP (NO_3_)) was used, where nitrate was substituted instead of ammonium as the sole nitrogen source to induce the artificial microRNA.

### 2.2 Generation of atx1 KO strains using CRISPR/LbCpf1

CC‐425, a cell wall reduced arginine auxotrophic strain, was used for transformation with an RNP complex consisting of a gRNA targeting a PAM sequence in intron1 of *ATX1* and LbCpf1 as described in Ferenczi et al. (2017) and shown in Figure 5 with the following modifications: Cells were grown to a density of 2 × 10^6^ cells per milliliter and counted using a Coulter counter. Cells (2 × 10^7^) were collected by centrifugation (5 min, 1500 *g*) and resuspended twice in Max Efficiency Transformation Reagent (1 ml), followed by resuspension in 230 μl of the same reagent supplemented with sucrose (40 mM). Cells were incubated at 40°C for 20 min. Purified LbCpf1 (80 μM) was preincubated with gRNA (1 nmol, targeting TTTGCGCCGCCCGCAGTGTCCAACG) at 25°C for 20 min to form RNP complexes. For transfection, 230 μl cell culture (2 × 10^7^ cells) was supplemented with sucrose (40 mM) and mixed with preincubated RNPs and HindIII digested pMS666 containing the *ARG7* gene, which complements the defective *arg7* gene in strain CC‐425 and thus confers the ability to grow without arginine supplementation in the medium. In order to achieve template DNA‐mediated editing, single‐stranded oligodeoxynucleotide (ssODN) (4 nmol, sequence containing two stop codons within exon 2 after the PAM target site) (GGGGCGGGAGTTGGACACAATCTCAATAGCCTACGTTGCACCCCTTTGCGCCGCCCGCAGTTAATAGCGTGTCCTGGGAAAGCTGGATGGAGTGGACTCGTACGAGGTCAGCTTGGAGAA) was added (Figure 5b). The final volume of the transformation reaction was 280 μl. Cells were electroporated in a 4‐mm gap cuvette (Bio‐Rad) at 600 V, 50 μF, 200 Ω by using Gene Pulser Xcell (Bio‐Rad). Immediately after electroporation, cells were allowed to recover overnight in darkness without shaking in 5 ml TAP with 40 mM sucrose and 0.4% (w/v) polyethylene glycol 8000 and then plated after collection by centrifugation (5 min at RT and 1650 *g*) using the starch embedding method (with 60% corn starch). After 12 d, a total of 49 colonies were transferred to new plates. The following program was used for all colony PCR reactions using the Bio‐Rad iTAQ SYBR mastermix: 95°C for 5 min followed by 40 cycles of 95°C for 15 s, and 65°C for 60 s. A first colony PCR using oligos ATX1screenfor TTGCGCCGCCCGCAGTTAATAG and ATX1seqrev CACTCCTGGCAAAAGCACAG identified candidate strains that might have been successfully edited at the ATX1 locus (which will anneal and hence amplify). Clones that showed successful PCR amplification were screened a second time using oligos ATX1seqfor TTGGCGCGTAAGTAATGGTG and ATX1seqrev CACTCCTGGCAAAAGCACAG, and amplicons were sequenced using oligo ATX1seqrev, which revealed that we had ssODN‐mediated gene editing within the second exon of *ATX1* in two clones (out of 49) that showed introduction of the two in‐frame stop codons (Figure 5b).

### 2.3 RNA extraction and quantitative real‐time PCR

3 × 10^7^ cells were collected by centrifugation for 5 min at 1424 × *g*, 4°C. RNA was extracted using TRIzol reagent. For subsequent DNaseI treatment and cleanup of all RNA samples, we used the Zymo Research RNA Clean & Concentrator™‐5 Kit according to the manufacturer's instructions. Reverse transcription was primed with oligo dT(18) using 2.5 μg of total RNA and SuperScript III Reverse Transcriptase (Invitrogen) according to the manufacturer's instructions. The subsequent cDNA was diluted 10‐fold before use. qRT‐PCR reactions contained 5 μl of cDNA corresponding to 100 ng of total RNA, 6 pmol of each forward and reverse oligonucleotide, and 10 μl of iTAQ Mastermix in a 20‐μl volume. The following program was used for all qRT‐PCR reactions: 95°C for 5 min followed by 40 cycles of 95°C for 15 s and 65°C for 60 s. Fluorescence was measured at the end of each 65°C cycle. A melting curve analysis was performed at the conclusion of the cycles from 65 to 95°C with fluorescence reads every 0.5°C. The abundance of *RACK1* served as reference transcript.

### 2.4 Antibody production and protein analyses

Antibodies targeting ATX1 were produced by Covance, Inc. by immunization using the subcutaneous implant procedure for rabbits on a 118‐day protocol with synthetic peptide Ac‐GVDSYEVSLEKQQAVVRGKALDPQAC‐amide. SDS‐polyacrylamide gel electrophoresis (PAGE) of total cell extracts or soluble protein fractions was performed using 20–40 μg of protein for each lane as indicated and transferred in a semidry blotter to nitrocellulose membranes (Amersham Protran 0.1 NC). The membrane was blocked for 30 min with 3% dried milk in PBS (137 mM NaCl, 2.7 mM KCL, 10 mM Na_2_HPO4, 1.8 mM KH_2_PO_4_) containing 0.1% (w/v) Tween 20 and incubated in primary antiserum; this solution was used as the diluent for both primary and secondary antibodies for 1 h, respectively. PBS containing 0.1% (w/v) Tween 20 was used for washing membranes twice for 15 min each time. The secondary antibody, used at 1:6000, was goat anti‐rabbit conjugated to alkaline phosphatase. Antibodies directed against ATX1 (1:500) and GFP (1:4000, Agrisera AS18 4227) were used as indicated.

### 2.5 YFP plasmid construct and cloning

For N‐terminal YFP tagging, we used the pRMB12 plasmid, which was a gift from R. Bock (MPI for Molecular Plant Physiology, Potsdam‐Golm) (Barahimipour et al., 2015). The *ATX1* gene (omitting the start codon ATG) was PCR amplified (Phusion Hot Start II polymerase, Thermo Fisher Scientific) from genomic DNA, gel purified (MinElute Gel Extraction Kit, QIAGEN), and cloned in‐frame with an N‐terminal Venus tag by Gibson assembly into EcoRI‐cut pRMB12 to generate pDS35. Primer sequences also introduced a glycine–serine (GS) linker between the *ATX1* sequence and YFP and were nATXfor: cctgggcatggacgagctgatcaagGGTGGTGGCGGTTCTTCTACCGAGGTGGTCCTTA and nATXrev: GTACAGGCGGTCCAGCTGCTGCCAGTTACGAGGACACGAGCTCGGCCTTCTTCCC.

For C‐terminal YFP tagging, we used the pLM005 plasmid (Mackinder et al., 2016). The ATX1 gene (omitting the stop codon TAA) was PCR amplified (Phusion Hot Start II polymerase, Thermo Fisher Scientific) from genomic DNA, gel purified (MinElute Gel Extraction Kit, QIAGEN), and cloned in‐frame with a C‐terminal Venus tag by Gibson assembly into HapI‐cut pLM005 to generate pDS46. Primer sequences were cATX1for: cactgctactcacaacaagcccagttATGTCTACCGAGGTGGTCCTTAA and cATX1rev: cgccggagccacccagatctccgttCGAGGACACGAGCTCGGCCTT.

pDS35 was linearized using XbaI, and pDS46 and pRMB12 were linearized with SpeI before transformation into UVM11 (a UV‐induced mutant derived from CC4350 [*cw15 arg7‐8 mt*+] known to efficiently express nuclear transgenes [Neupert et al., 2009] that was kindly provided by R. Bock [MPI for Molecular Plant Physiology, Potsdam‐Golm] using the glass bead method) (Kindle et al., 1989). Transformants were selected on 10 μg/μl paromomycin and screened by immunodetection using anti‐GFP (Agrisera AS18 4227).

For live cell imaging of all YFP‐expressing lines, cells were imaged within 1 h after cell collection on microscope slides on a Zeiss LSM 880 using 514 Ex and 527‐nm emission. Image processing was performed using the Zeiss ZEN software.

### 2.6 Quantitative metal, phosphorus, and sulfur content analysis

1 × 10^8^ cells (culture density of 3–5 × 10^6^ cells/ml) were collected by centrifugation at 2450 *g* for 3 min in a 50‐ml Falcon tube. The cells were washed two times in 50 ml of 1 mM Na_2_‐EDTA pH 8 (to remove cell surface‐associated metals) and once in Milli‐Q water. The cell pellet was stored at −20°C before being overlaid with 286 μl 70% nitric acid and digested at room temperature for 24 h and 65°C for about 2 h before being diluted to a final nitric acid concentration of 2% (v/v) with Milli‐Q water. Metal, sulfur, and phosphorus contents were determined by inductively coupled plasma mass spectrometry on an Agilent 8900 Triple Quadrupole instrument by comparison to an environmental calibration standard (Agilent 5183‐4688), a sulfur (Inorganic Ventures CGS1), and a phosphorus (Inorganic Ventures CGP1) standard. 89Y served as an internal standard (Inorganic Ventures MSY‐100PPM). The levels of analytes were determined in MS/MS mode. ^63^Cu analytes were measured directly using He in a collision reaction cell. ^56^Fe was directly determined using H_2_ as a cell gas. An average of four technical replicate measurements was used for each individual biological sample. The average variation between technical replicate measurements was less than 2% for all analytes and never exceeded 5% for an individual sample. Triplicate samples (from independent cultures) were also used to determine the variation between cultures. Averages and standard deviations between these replicates are depicted in figures.

## 3 RESULTS

### 3.1 ATX1 is conserved in most algae

Because the inventory of cupro‐proteins in algae is distinct from that in other eukaryotes (yeast, human, land plants), we sought to understand the role of the Chlamydomonas orthologue of the ATX1 copper chaperone. We recovered ATX1 sequences from various eukaryotic organisms using yATX1 as a query (Figure 1). A homologue was found in most algae, indicating the selective pressure for maintaining this molecule even if some algae can grow in Cu‐deficient medium by dispensing with abundant Cu proteins (Blaby‐Haas & Merchant, 2017; Merchant et al., 2020). Generally, the yATX1 homologue occurs as a single copy gene, which is in contrast to land plants where at least two distinct ATX1 homologues are found in addition to the Cu chaperones for Cyt oxidase and superoxide dismutase, Cox17 and Ccs1, respectively (Himelblau et al., 1998; Puig et al., 2007). All algal ATX1 proteins share the MXCXXC domain that is required for Cu binding and a defining characteristic of the ATX1 family of Cu chaperones (Supplemental Figure 1).

### 3.2 ATX1 is predominantly expressed in conditions in which iron is scarce

**FIGURE 2:**
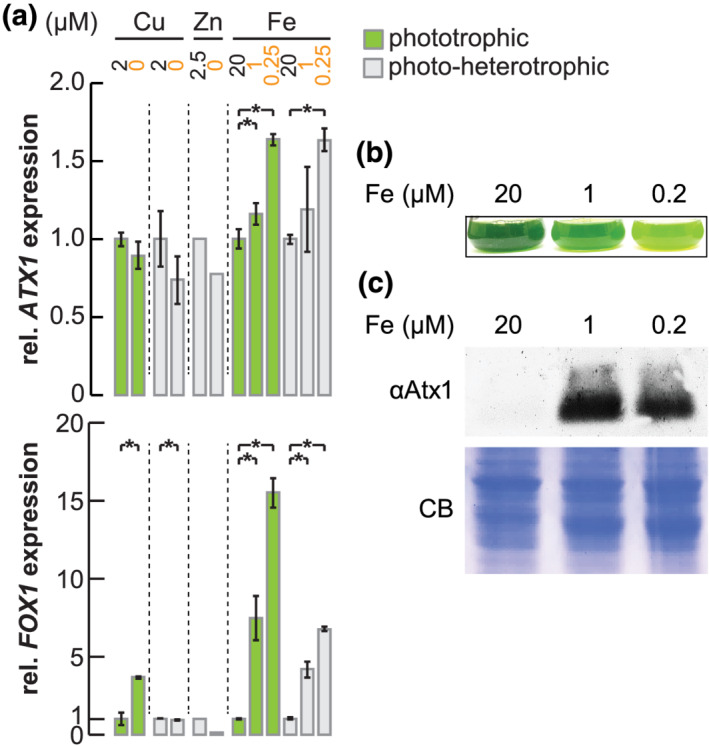
Atx1 expression depends on iron nutritional status. (a) Relative abundance changes of *ATX1* (upper panel) and *FOX1* (bottom panel) transcripts upon trace metal limitation (Cu, Zn, or Fe). Horizontal lines separate different experiments. Green bars indicate experiments under phototropic conditions; gray bars indicate presence of acetate in the light. Shown are averages and standard deviation from at least two independent replicates. Asterisks indicate significance (*t*‐test, α ≤  0.05). Data are reanalyzed from Castruita et al. (2011), Malasarn et al. (2013), and Urzica et al. (2012). (b) *Chlamydomonas reinhardtii* cells CC‐4533 were grown in either iron‐replete TAP medium (20), in Fe‐deficient (1), or in Fe limited (0.2) growth medium as indicated. A picture of flasks was taken 5‐day post‐inoculation. (c) Total soluble protein was separated by 15% SDS PAGE and immunodetected using antisera against ATX1. Coomassie blue (CB) stain was used as loading control. Shown is one of two experiments performed with independent cultures

Based on the sequence relationship of CrATX1 with the yeast and *Arabidopsis* proteins and the parallel increases in *ATX1* and *FOX1* transcript abundances under low iron conditions (La Fontaine et al., 2002), we hypothesized that CrATX1 functioned in Cu delivery to the multicopper oxidase via the secretory pathway as in other organisms. A survey of *ATX1* expression in large‐scale RNA‐seq datasets from experiments involving Fe, Cu, and Zn depletion (Castruita et al., 2011; Hong‐Hermesdorf et al., 2014; Urzica et al., 2012) confirmed that *ATX1* is responsive to poor iron nutrition (Figure 2a). Indeed, transcripts increase already under asymptomatic Fe deficiency and are further increased in the Fe‐limited symptomatic state. The pattern of expression is unaffected by carbon source (CO_2_ vs. acetate) nor by other trace nutrient deficiencies like Cu or Zn. In general, a survey of 56 RNA‐sequencing datasets indicated that *ATX1* transcript abundances are quite stable across various nutrient regimes and other stress conditions (Supplemental Figure 2), with Fe and nitrogen nutrition being notable exceptions. *FOX1* transcripts, on the other hand, are increased not only in Fe deficiency but also in Cu‐deficient conditions and are reduced in Zn‐deficient cells (Figure 2a). *ATX1* and *FOX1* are therefore not strictly co‐expressed; simultaneous expression is limited to conditions involving reduction of Fe in the growth medium.

To test the impact of the change in *ATX1* mRNA abundance, we analyzed the abundance of the corresponding polypeptide as a function of iron nutrition. Chlamydomonas cells were grown in TAP medium supplemented with 20, 1, and 0.2 μM iron, corresponding to replete, deficient, and limited growth, respectively (Glaesener et al., 2013). We recapitulated previously reported phenotypic differences in growth, including chlorosis and growth arrest on media containing 1 and 0.2 μM Fe as compared with 20 μM Fe (Figure 2b). Chlorosis was particularly strong in iron‐limited cultures, likely due to complete exhaustion of the iron source from the growth medium (Page et al., 2012). Immunoblot analysis indicated that ATX1 is iron‐conditionally expressed (Figure 2b), which is consistent with a role for Chlamydomonas ATX1 in Cu delivery to the secretory pathway for FOX1 biosynthesis.

### 3.3 ATX1 is a cytosolic protein

**FIGURE 3:**
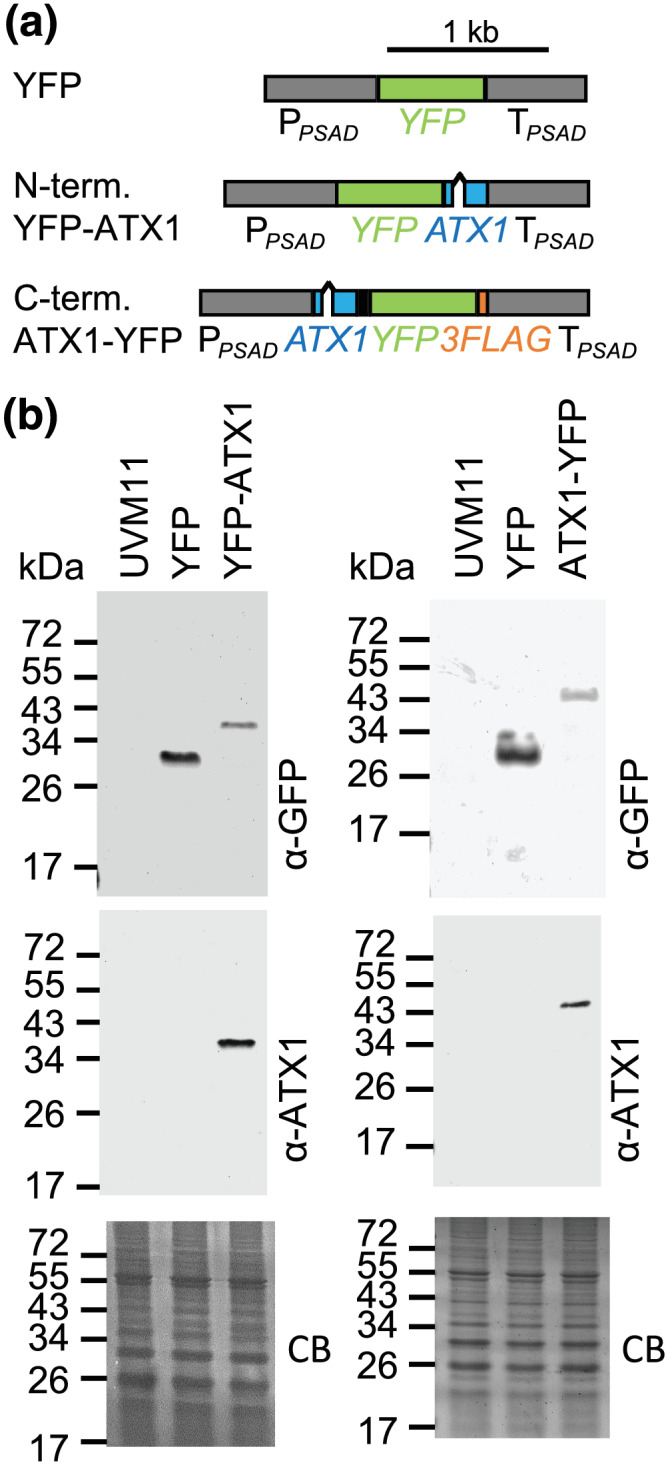
YFP‐Atx1 is expressed in *Chlamydomonas*. (a) Physical map of the YFP control, the YFP‐ATX1, and the ATX1‐YFP construct. (b) Total protein from UVM11 (parental strain) and UVM11 transformed strains expressing either the N‐terminal YFP‐ATX1 fusion protein or the C‐terminal ATX1‐YFP fusion protein as well as YFP were separated by 15% SDS PAGE and after transfer to a nitrocellulose membrane probed using a GFP antibody. ATX1 antibody was also tested in the assay to confirm cross‐reactivity with the fusion protein. Coomassie blue stain (CB) of the gel is shown as a loading control. The expected size of YFP is 26.7 kDa, of the N‐terminal YFP‐ATX1 fusion protein is 34.2 kDa, and of the C‐terminal ATX1‐YFP fusion protein is 40.1 kDa

**FIGURE 4:**
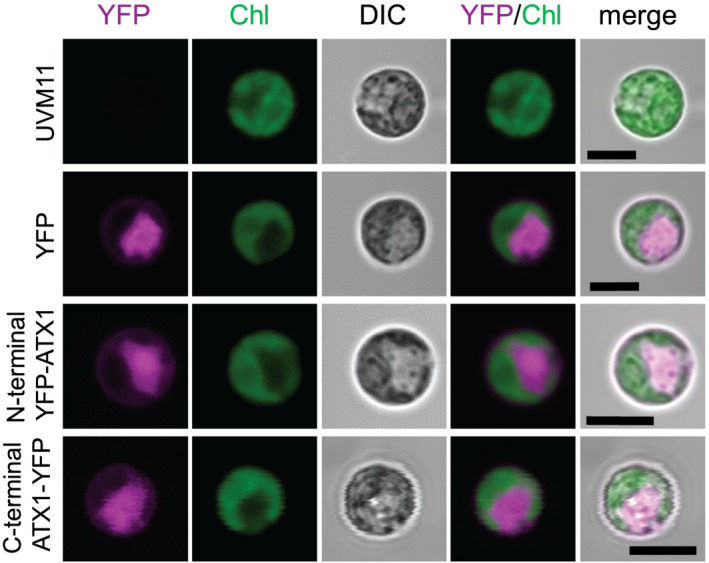
Subcellular localization of YFP, an N‐terminal YFP‐ATX1 fusion protein as well as a C‐terminal ATX1‐YFP fusion protein. Confocal microscopy of UVM11 control (untransformed), ATX1 protein fused with YFP at the N‐or C‐terminus (magenta), and YFP expressed without a fusion protein driven by the constitutive *PSAD* promoter, respectively. Chlorophyll autofluorescence is shown in green (Chl) and bright‐field images (DIC) are shown to depict the cell and the chloroplast, respectively. All strains were grown in medium supplemented with 1 μM Fe. Scale bars correspond to 5 μm. At least 10 individual cells derived from two independent cultures were imaged and are shown in Supplemental Figures 3–6

To distinguish ATX1 localization, we tagged the protein with a fluorescent reporter (YFP) at the N‐terminus and at the C‐terminus and expressed the respective fusion proteins from a strong, constitutive promoter (*PSAD*; Figure 3a) into strain UVM11, which expresses nuclear transgenes efficiently (Neupert et al., 2009). We identified a line for each of the constructs that showed a signal against both ATX1 and GFP antiserum with a band in each case at the expected size of the fusion protein (Figure 3b). The analysis using the GFP antiserum showed no other visible bands below the size of the fusion protein, indicating that there were no cleavage products and no expression of YFP without ATX1 in the transformants, which would potentially interfere with localization analysis using confocal microscopy (Figure 3b). We chose a line expressing YFP alone, the abovementioned line expressing an N‐terminal YFP‐ATX1 fusion protein, as well as a line expressing a C‐terminal ATX1‐YFP fusion protein (Figure 3) for further analyses by direct live cell imaging. Notably, using chlorophyll autofluorescence to visualize the chloroplast, we conclude that both YFP‐ATX1 fusion proteins are localized to the cytoplasm, consistent with ATX1's proposed role in Cu trafficking from the inner plasma membrane to the trans‐Golgi network (Figure 4).

### 3.4 ATX1 is required for growth in low iron conditions

**FIGURE 5:**
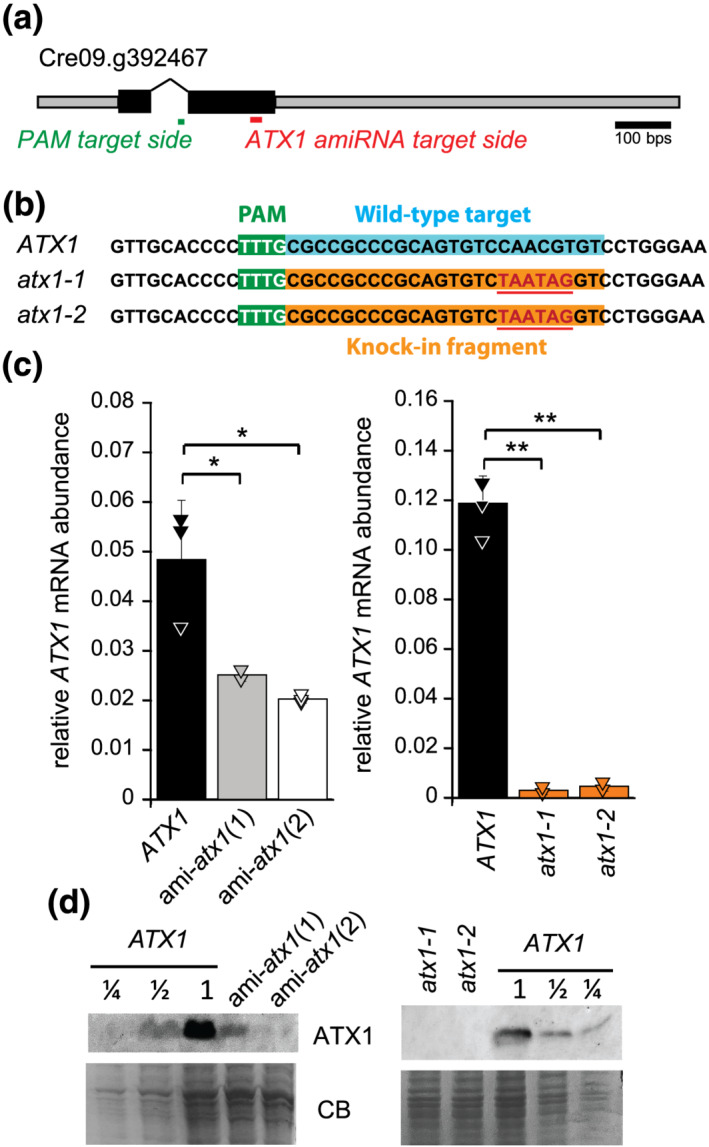
Strains with reduced ATX1 abundance using artificial microRNA constructs and *atx1* KO lines using CRISPR/Cpf1‐mediated gene editing. (a) Physical map of the *ATX1* gene and target site for the amiRNA as well as the PAM sequence for Cpf1 recruitment. (b) CRISPR/Cpf1‐ and ssODN‐mediated gene editing resulted in the insertion of two in‐frame stop codons into *ATX1*. (c) amiRNA lines 1, 2, *atx1‐1*, and *atx1‐2* grown in TAP supplemented with 0.2 μm Fe (limited) showed reduced *ATX1* mRNA abundance in comparison with the respective Fe‐limited reference strains (*ATX1*). (d) Soluble protein from Fe‐limited cultures was separated by 15% SDS‐PAGE, transferred to nitrocellulose membranes, and immunodetection was performed using antisera against ATX1 to confirm reduced ATX1 protein abundance in *atx1* amiRNA lines and absence of ATX1 protein in *atx1* mutants. Coomassie blue (CB) stain was used as loading control

To validate the function of ATX1 in *Chlamydomonas*, we designed an artificial microRNA (amiRNA) targeting the 3′ coding sequence of the Cr*ATX1* gene (Figure 5a, red bar) (Molnár et al., 2009; Schmollinger et al., 2010). We initially screened candidate *ATX1* knockdown lines based on reduction of *ATX1* mRNA as compared with control lines containing an empty vector that lacks the target region against CrATX1 (*ATX1*). The candidate knockdown lines were grown in 0.2 μM Fe to maximally induce *ATX1* expression to improve signal to noise in the screen. We chose two lines that showed reduced *ATX1* mRNA abundance, which we named ami‐*atx1*(1) and ami‐*atx1*(2) (Figure 5c). Compared with empty vector‐transformed control strains, the abundance of ATX1 was reduced to about 50% and 25% in Fe‐limited ami‐*atx1*(1) and ami‐*atx1*(2), respectively (Figure 5c). In parallel, we generated *atx1* knockout lines using CRISPR/CPF1‐mediated gene editing adapted from previous protocols using homology‐directed DNA replacement and the enzyme LbCpf1 in combination with a selection marker (Ferenczi et al., 2017; Greiner et al., 2017). *ATX1* has a PAM (protospacer adjacent motif) target sequence for LbCPF1 recruitment within its intron (Figure 5, green bar) that allowed gene editing at the downstream Cpf1 cleavage site within the second exon of *ATX1*. We used ssODNs as a repair template to introduce two in‐frame stop codons within the second exon of the *ATX1* gene (Figure 5b). Sequencing of the PCR product spanning the gene editing site confirmed the presence of the two stop codons in two *atx1* lines, depicted *atx1‐1* and *atx1‐2* (Figure 5b).

**FIGURE 6:**
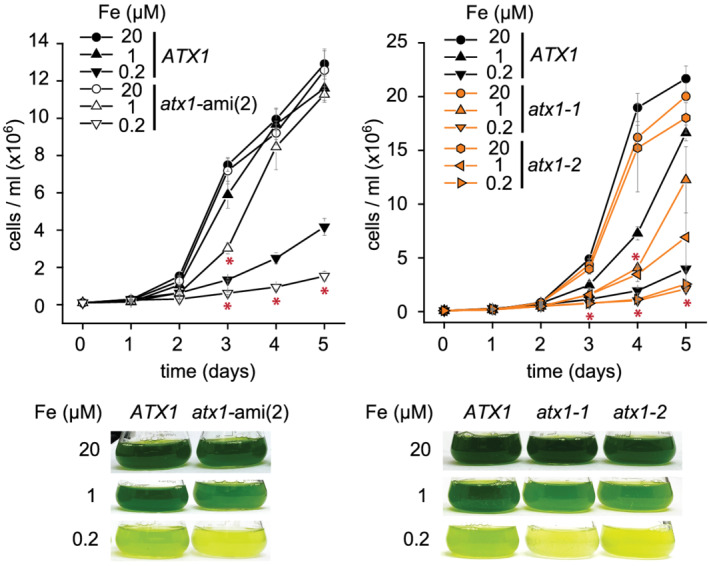
Iron nutrition‐dependent growth defect is exacerbated in *ATX1, atx1*‐ami lines and *atx1* mutants. *ATX1* reference lines (black, filled symbols), *atx1*‐ami(2) (white symbols), and *atx1* mutants (orange‐filled symbols) were grown in either iron‐replete TAP medium (20), in Fe‐deficient (1), or in Fe‐limited (0.2) growth medium as indicated. Cell counts were obtained at 24‐h intervals. Shown are averages and standard deviation from three independent grown cultures. Below each panel are pictures of flasks taken 4‐days post‐inoculation. Asterisks indicate significant changes between *atx1*‐ami lines and reference strains or between both *atx1* mutants and respective reference strains at the same time point, respectively. Significant differences were determined by one‐way ANOVA followed by Holm–Sidak, *p*‐value  α ≤  0.05

**FIGURE 7:**
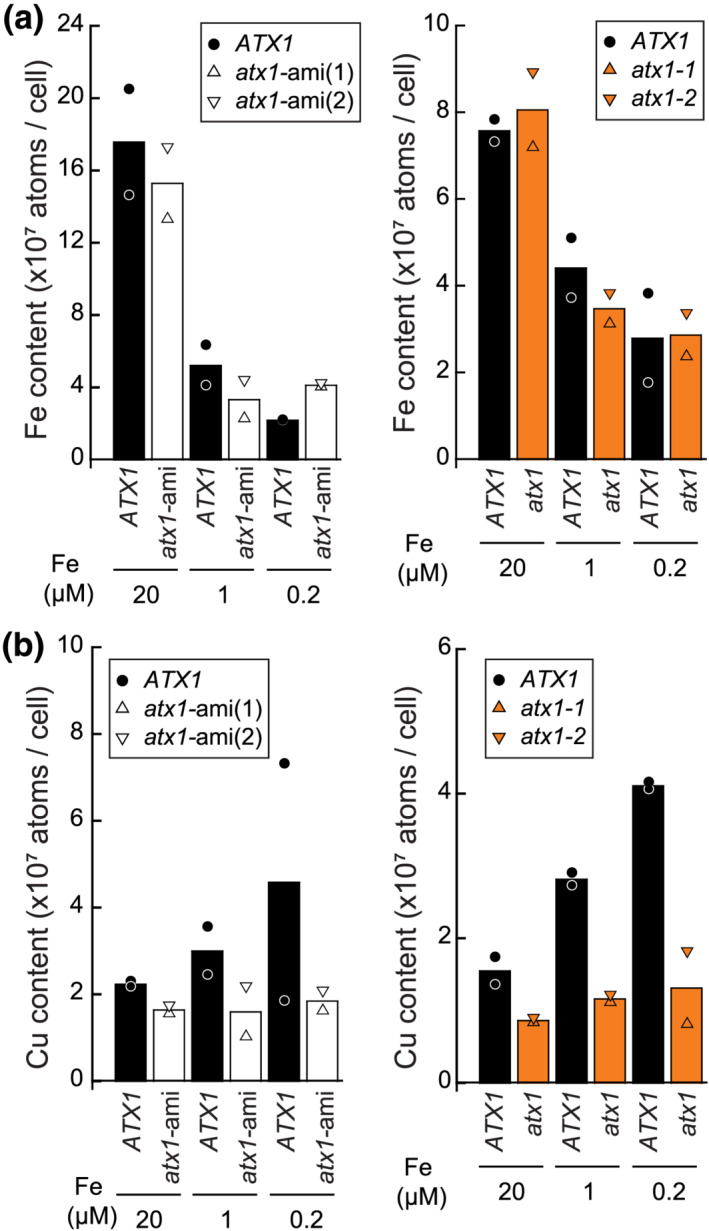
Iron and copper content in *atx1* mutants and *atx1*‐amiRNA lines. (a) Iron and (b) copper content of empty vector (*ATX1*, black) and *atx1*‐ami lines (*atx1*‐ami(1) and *atx1*‐ami(2), white triangles), mutant background strains (ATX1, black), *atx1* mutants (*atx1‐1* and *atx1‐2*, orange triangles) that were grown in media containing iron as indicated (replete 20, deficient 1, and limited 0.2) was measured by ICP‐MS. Shown are averages and individual values from independent grown cultures

If ATX1 functions in Cu trafficking toward the secretory pathway, *atx1* mutant lines would be affected in FOX1 metalation and hence display a phenotype in iron‐poor medium where FOX1 function is required (Terzulli & Kosman, 2009). To test this, we grew ami‐*atx1*(2), *atx1‐1*, and *atx1‐2* and corresponding control lines in iron‐replete, iron‐deficient, and iron‐limiting media. Neither the amiRNA line nor *atx1* mutants showed a growth defect in iron replete conditions, which is expected because ATX1 and its proposed client FOX1 are barely expressed in this situation (Figure 6). On the other hand, in iron‐limiting conditions, both ami‐*atx1*(2) and *atx1* mutants showed reduced growth as compared with control lines (Figure 6). When we measured the Fe content of each strain by ICP‐MS/MS, we noted that all strains showed a reduction of the Fe content down to a basal (presumed essential) level in Fe‐poor conditions. Because Fe is a growth‐limiting nutrient, Fe‐limited cells cease growth when the basal essential level of iron cannot be supported. Accordingly, *atx1* mutant and ami‐*atx1* lines have reduced growth but maintain the minimal essential Fe quota comparable with wild‐type lines (Figure 7a).

We noticed before that Fe‐deficient cells slightly upregulate components of the Cu assimilation pathway, likely due to a link between both pathways via FOX1 (Dancis et al., 1994). Accordingly, the copper content of cells grown on growth medium lacking iron is increased twofold compared with iron‐replete grown cells (Figure 7b). On the other hand, Cu content was significantly lower in ami‐*atx1* lines as well as in *atx1* mutants as compared with their respective reference lines in all conditions that were analyzed (Figure 7b). These data indicate a second phenotype in *atx1* mutants, namely, a remarkable impact on Cu assimilation and accumulation.

### 3.5 ATX1 is important for purine assimilation in nitrogen‐poor conditions

**FIGURE 8:**
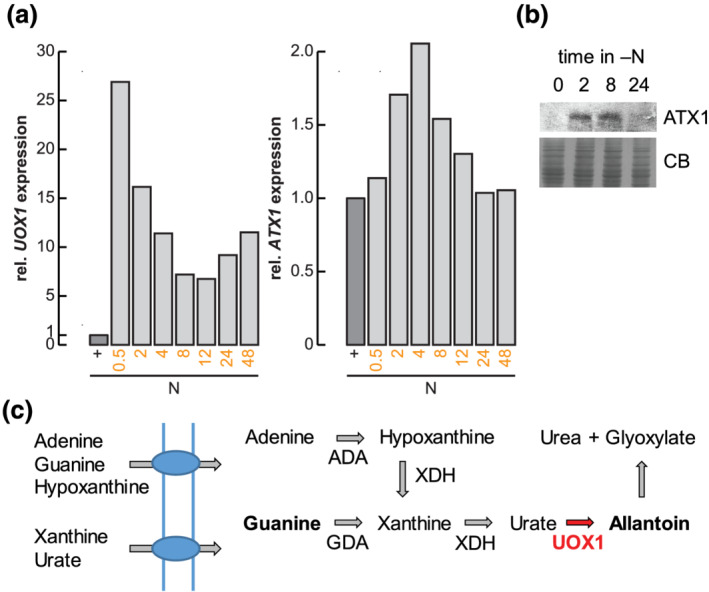
*UOX1* and *ATX1* are transiently expressed upon transfer to N‐free medium. (a) Relative abundance changes of *UOX1* (left panel) and *ATX1* (right panel) transcripts upon nitrogen limitation. Data are reanalyzed from Boyle et al. (2012) and Schmollinger et al. (2014). (b) Soluble protein from N‐limited cultures was separated by 15% SDS‐PAGE, transferred to nitrocellulose membranes, and immunodetection was performed using antiserum against ATX1. Coomassie blue (CB) stain was used as loading control. Shown is one of two independent experiments. (c) Shown is the nitrogen assimilation pathway from purines toward urea. AC, allantoicase; ADA, adenine deaminase; GDA, guanine deaminase; UOX1, urate oxidase; XDH, xanthine dehydrogenase

**FIGURE 9:**
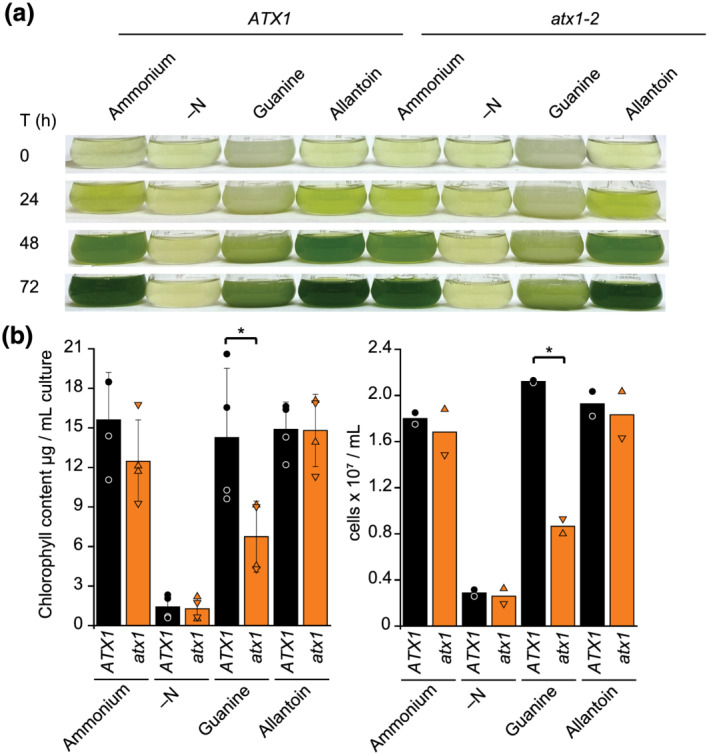
Guanine‐dependent growth defect in *atx1* mutants. (a) Reference lines and *atx1* mutants were grown in medium lacking any nitrogen source (–N) or supplemented with ammonium, guanine, or allantoin as sole nitrogen source as indicated. Pictures of flasks were taken in 24‐h intervals. (b) Shown is the chlorophyll content (left panel) and cell concentration (right panel) of all strains and conditions at time 72 h. Shown are averages, standard deviation, and individual values from independent grown cultures. Mutant background strains are depicted as *ATX1*, black circles; the *atx1–1* mutant is depicted as orange triangle up and the *atx1‐2* mutant as orange triangle down. Asterisks indicate significant changes determined by one‐way ANOVA followed by Holm–Sidak, *p*‐value ≤  0.05

Urate oxidase, an enzyme involved in purine metabolism (Figure 8), is also a Cu enzyme, making it another candidate client of ATX1 (Alamillo et al., 1991). In accordance with our expectations, we noted that *UOX1*, encoding urate oxidase, and *ATX1* transcripts both increase transiently under N deficiency (Figure 8a). Notably, the observed increase in *ATX1* transcript abundance upon N starvation is paralleled by an increase in the corresponding polypeptide (Figure 8b). To test whether urate oxidase is an ATX1 client, we grew the reference wild‐type strains and each *atx1* mutant on guanine versus allantoin as a sole nitrogen source (Figure 8c). Both guanine and allantoin are taken up by Chlamydomonas, but guanine metabolism requires UOX1 whereas allantoin metabolism does not (Lisa et al., 1995; Merchán et al., 2001; Piedras et al., 1998). Strikingly, after 72 h of growth, *atx1* mutants showed a visible growth defect on guanine but not allantoin as compared with the corresponding reference lines (Figure 9), with significant less biomass accumulation as determined by chlorophyll content and cell counts (Figure 9b). We conclude that ATX1 is required for UOX1 function, most likely for its metalation.

## 4 DISCUSSION

### 4.1 CrATX1: A route for Cu toward the secretory pathway?

In Chlamydomonas, multiple pathways of Cu trafficking emerge from the high‐affinity, CTR‐type Cu import system (Page et al., 2009). Cu(I) taken up by CTRs is delivered to cytosolic Cu chaperones, perhaps via glutathione as an intermediate carrier (Miras et al., 2008), for redistribution to the mitochondria, to the chloroplast, and to the secretory pathway (Valentine & Gralla, 1997). The work presented herein describes a soluble Cu chaperone in Chlamydomonas, CrATX1, that we propose functions in Cu delivery to enzymes that are processed in the secretory pathway. Several lines of evidence support a role for CrATX1 in Cu sequestration from the inner surface of the plasma membrane region to the secretory pathway, where Cu is subsequently provided to the multicopper Fe(II) oxidase FOX1, the urate oxidase UOX1, and perhaps other secreted Cu containing target proteins awaiting maturation and metalation.

First, both FOX1 and ATX1 are conditionally expressed as a function of iron nutrition. Cells grown in iron‐limited media are characterized by chlorosis and growth arrest, and this phenotype is exacerbated in *atx1* mutants. Yet, strains with reduced or even no ATX1 abundance have the same iron content as do wild‐type cells. This phenotype mirrors that noted in *fox1* knockdown lines (Chen et al., 2008). This is because Fe is a growth‐limiting nutrient. When the minimal Fe quota is not met, no further growth can occur, and ami‐*atx1* lines as well as *atx1* mutants recapitulate the iron conditional growth defect of *fox1* knockdown lines. Because both *fox1* and *atx1* mutant strains do grow, albeit poorly, in iron‐poor conditions, we propose that there must be a Cu‐independent alternative system to facilitate iron uptake. The ZIP family transporters IRT1 and IRT2 are likely candidates (Blaby‐Haas & Merchant, 2012; Chen et al., 2008). This is further supported by the observation that Cu‐deficient cells are also not secondarily iron deficient, despite FOX1 lacking its Cu cofactor in this situation (Kropat et al., 2015).

Second, we see a growth phenotype when cells are grown in media with guanine as the sole nitrogen source. In this situation, cells are dependent on purine assimilation via UOX1, which is also metalated in the secretory pathway (Alamillo et al., 1991). Again, the *atx1* mutants show reduced growth as compared with reference wild‐type lines. The third line of evidence is the reduced copper content of *atx1* mutants, which speaks to its role in Cu(I) assimilation.

By expressing an N‐terminal and a C‐terminal YFP‐ATX1 fusion protein in *Chlamydomonas*, we were able to demonstrate that CrATX1 is localized within the cytoplasm, consistent with the function of ATX1 in other organisms. Chlamydomonas encodes several Cu‐dependent P‐type ATPases (Blaby‐Haas & Merchant, 2012), one of which, CTP1, is proposed to serve as the yeast Ccc2 orthologue, pumping Cu(I) into the lumen of the Golgi compartment. Like *ATX1*, *CTP1* expression is also induced in Fe‐starved cells (La Fontaine et al., 2002). This work documents that the loading of Chlamydomonas FOX1 and potentially also urate oxidase is dependent on this Cu(I) delivery pathway.

## CONFLICT OF INTEREST

The authors have no conflicts of interest to declare.

## Supporting information


**Supplemental Figure 1.** Shown is a multiple sequence alignment using Atx1 protein sequences from diverse organisms.
**Supplemental Figure 2.** Expression estimates for *ATX1* (Cre09.g392467) from 56 different RNAseq experiments. Data for Supplemental Figure 2 is reanalyzed from (Salomé and Merchant 2021).
**Supplemental Figure 3.** The N‐terminal YFP‐ATX1 fusion protein localizes to the cytosol. Figure shows all cells that were imaged in experiments shown and described in Figure 4.
**Supplemental Figure 4**. YFP localizes to the cytosol. Figure shows all cells that were imaged in experiments shown and described in Figure 4.
**Supplemental Figure 5.** No YFP signal was detected in the UVM11 background strain. Figure shows all cells that were imaged in experiments shown and described in Figure 4.
**Supplemental Figure 6.** The C‐terminal ATX1‐YFP fusion protein localizes to the cytosol. Figure shows all cells that were imaged in experiments shown and described in Figure 4.
