# Supplementary material for: Chlamydomonas ATX1 is essential for Cu distribution to multiple cupro‐enzymes and maintenance of biomass in conditions demanding cupro‐enzyme‐dependent metabolic pathways
Source: Plant Direct. 2022 Feb 3;6(2):e383. doi: 10.1002/pld3.383 (PMC8814560; doi:10.1002/pld3.383)
Supplement: Supplementary file 1 — Supplemental Figure 1. Shown is a multiple sequence alignment using Atx1 protein sequences from diverse organisms. Supplemental Figure 2. Expression estimates for ATX1 (Cre09.g392467) from 56 different RNAseq experiments. Data for Supplemental Figure 2 is reanalyzed from (Salomé and Merchant 2021). Supplemental Figure 3. The N‐terminal YFP‐ATX1 fusion protein localizes to the cytosol. Figure shows all cells that were imaged in experiments shown and described in Figure 4. Supplemental Figure 4. YFP localizes to the cytosol. Figure shows all cells that were imaged in experiments shown and described in Figure 4. Supplemental Figure 5. No YFP signal was detected in the UVM11 background strain. Figure shows all cells that were imaged in experiments shown and described in Figure 4. Supplemental Figure 6. The C‐terminal ATX1‐YFP fusion protein localizes to the cytosol. Figure shows all cells that were imaged in experiments shown and described in Figure 4. [file PLD3-6-e383-s001.pdf]

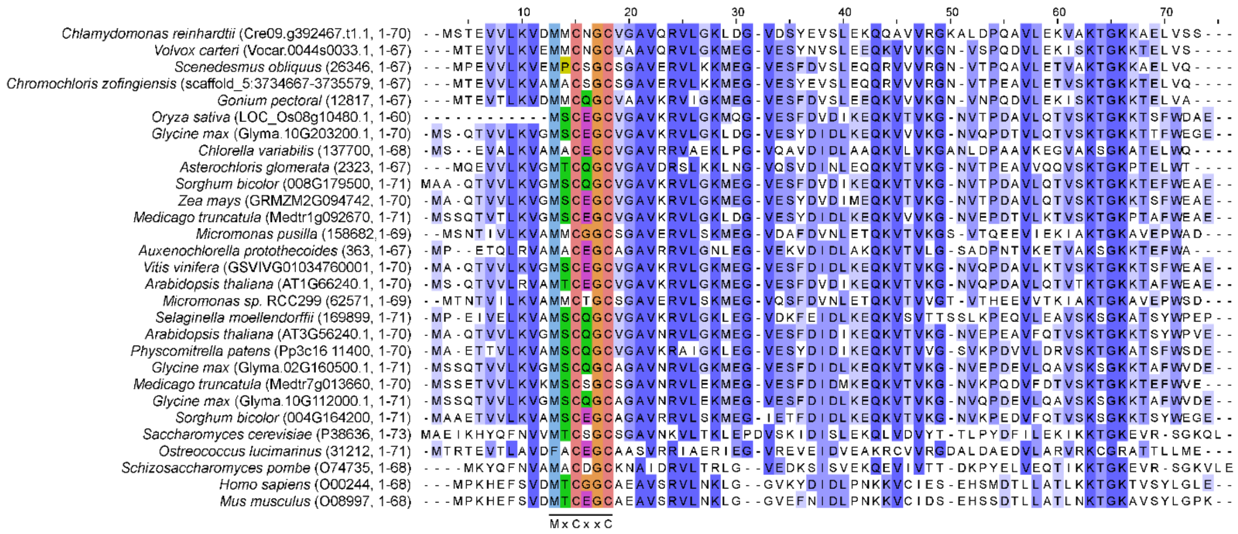

**Supplemental Figure 1**. Shown is a multiple sequence alignment using Atx1 protein sequences from diverse organisms.

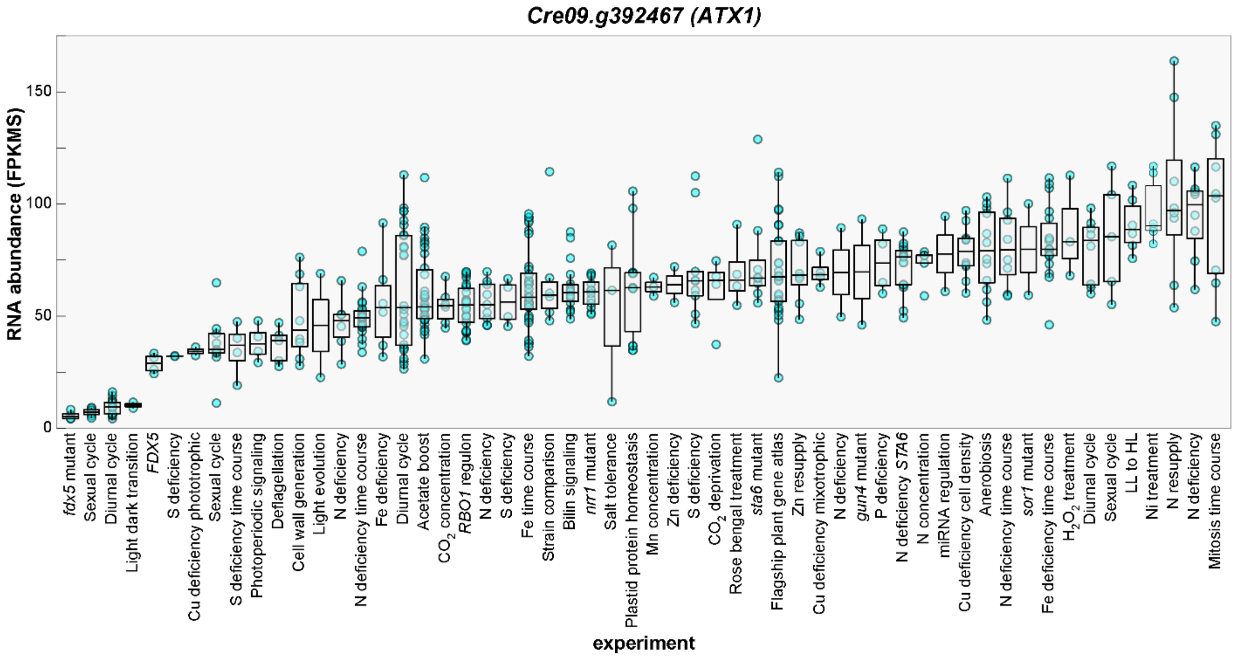


**Supplemental Figure 2.** Expression estimates for *ATX1* (Cre09.g392467) from 56 different RNAseq experiments. Data for Supplemental Figure 2 is reanalyzed from (Salomé and Merchant 2021).

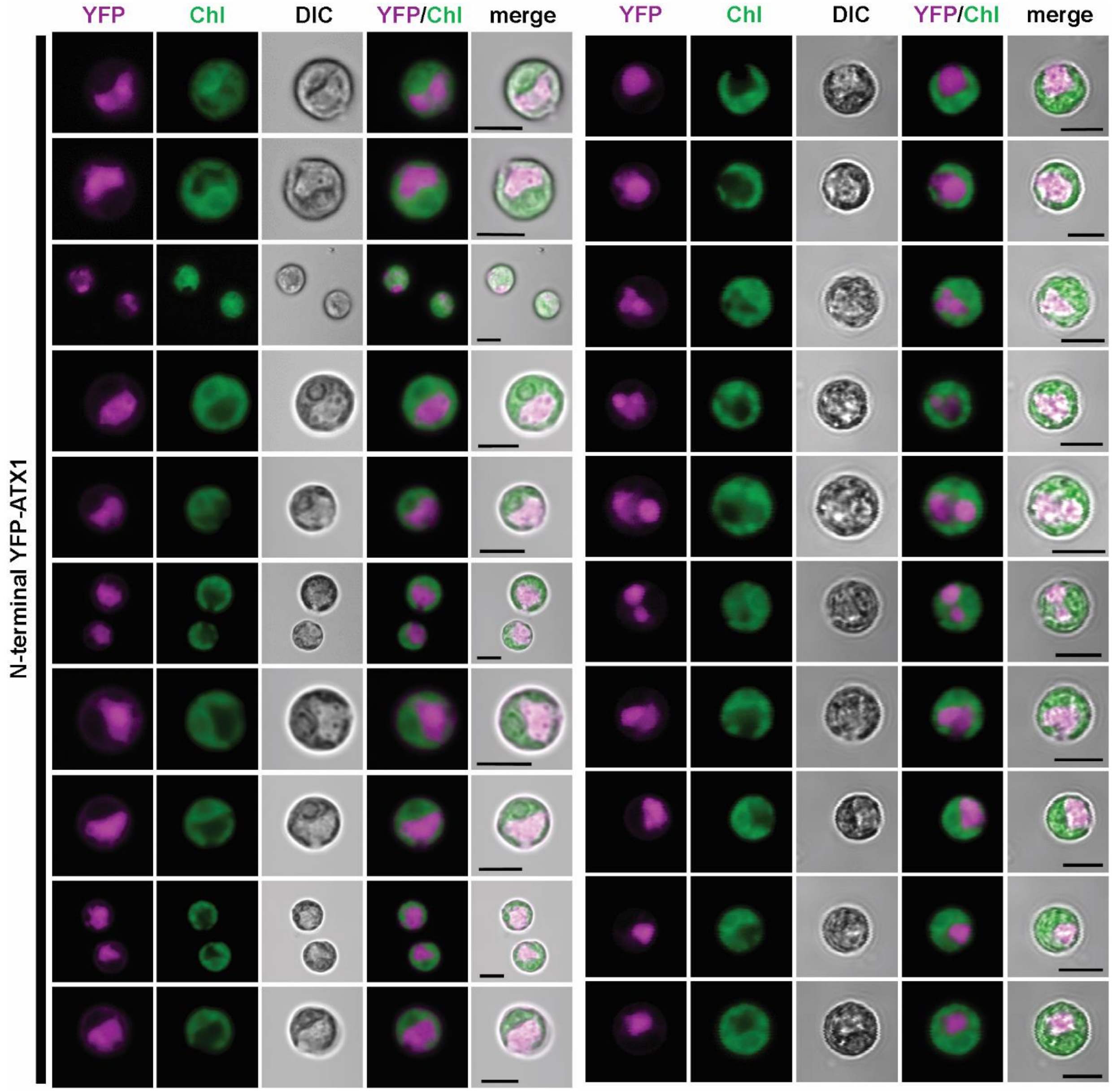


**Supplemental Figure 3.** The N-terminal YFP-ATX1 fusion protein localizes to the cytosol. Figure shows all cells that were imaged in experiments shown and described in Figure 4.

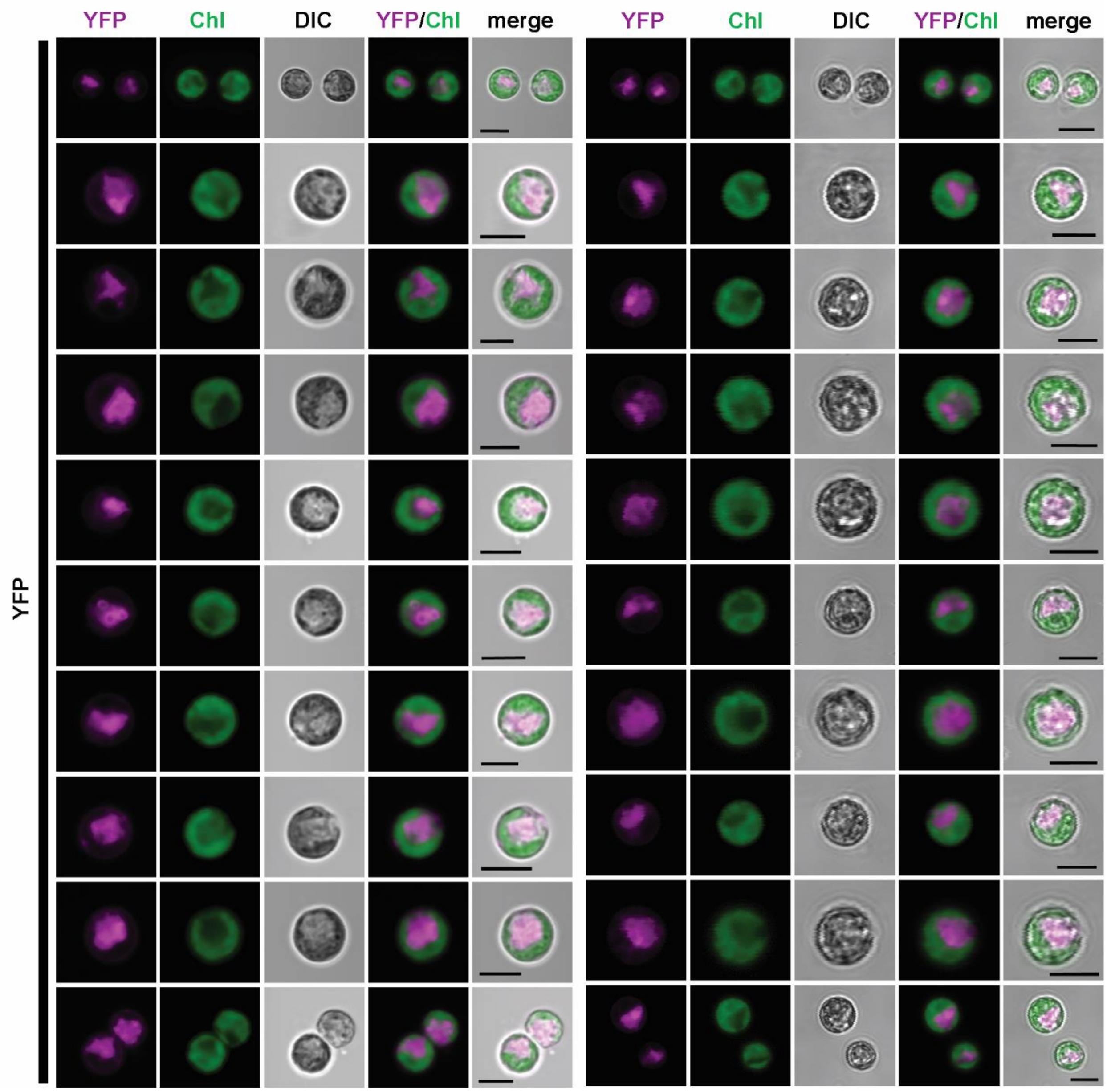


**Supplemental Figure 4**. YFP localizes to the cytosol. Figure shows all cells that were imaged in experiments shown and described in Figure 4.

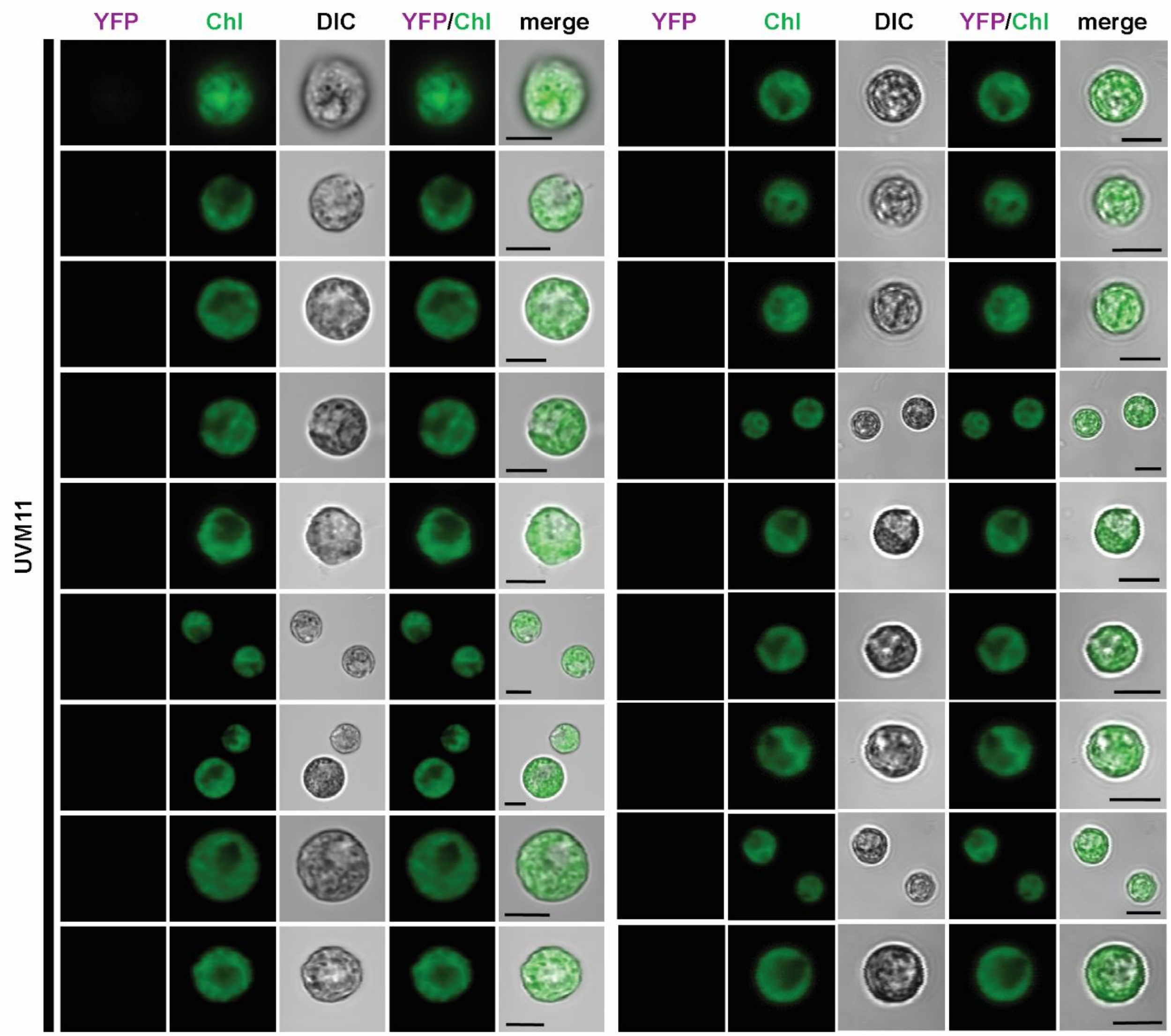


**Supplemental Figure 5.** No YFP signal was detected in the UVM11 background strain. Figure shows all cells that were imaged in experiments shown and described in Figure 4.

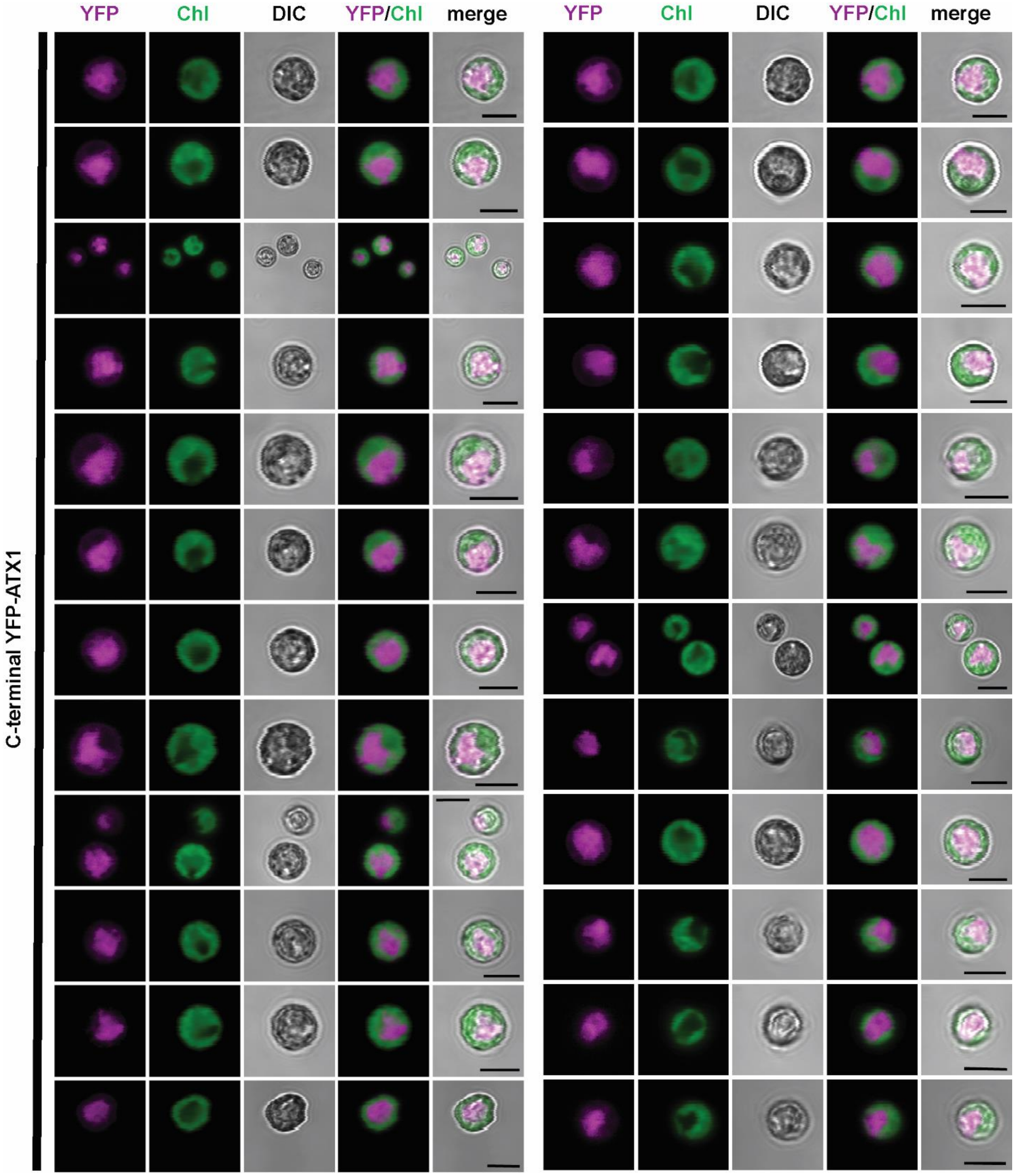


**Supplemental Figure 6.** The C-terminal ATX1-YFP fusion protein localizes to the cytosol. Figure shows all cells that were imaged in experiments shown and described in Figure 4.
